# Chemical Composition and Antioxidant Activity of the Essential Oils of Citral-Rich Chemotype *Cinnamomum camphora* and *Cinnamomum bodinieri*

**DOI:** 10.3390/molecules27217356

**Published:** 2022-10-29

**Authors:** Qingyan Ling, Beihong Zhang, Yanbo Wang, Zufei Xiao, Jiexi Hou, Changlong Xiao, Yuanqiu Liu, Zhinong Jin

**Affiliations:** 1 Key Laboratory of Silviculture, Co-Innovation Center of Jiangxi Typical Trees Cultivation and Utilization, College of Forestry, Jiangxi Agricultural University, Zhimin Rd. 1101, Nanchang 330045, China; 2 Jiangxi Provincial Engineering Research Center for Seed-Breeding and Utilization of Camphor Trees, School of Hydraulic and Ecological Engineering, Nanchang Institute of Technology, Nanchang 330099, China

**Keywords:** *Cinnamomum camphora*, *Cinnamomum bodinieri*, citral chemotype, essential oil, chemical composition, antioxidant capacity

## Abstract

Citral chemotypes *Cinnamomum camphora (C. camphora)* and *Cinnamomum bodinieri (C. bodinieri)* are promising industrial plants that contain abundant citral. For a more in-depth study, their significant biological effect, the chemical composition and antioxidant capacity of essential oils of citral-rich chemotype *C. camphora* and *C. bodinieri* (EOCC) were determined in the present study. The EOCC yield, obtained by hydro-distillation and analyzed by gas chromatography–mass spectrometry (GC-MS), ranged from 1.45–2.64%. Forty components more than 0.1% were identified and represented, mainly by a high content of neral (28.6–39.2%), geranial (31.8–54.1%), Z-isocitral (1.8–3.2%), E-isocitral (3.2–4.7%), geraniol (1.3–2.6%) and caryophyllene (0.6–2.4%). The antioxidant properties of EOCC were estimated by DPPH, ABTS and FRAP methods. As our results indicated, the antioxidant activity was significantly correlated to oxygenated monoterpenes. The variety of *C. bodinieri* (N7) presented the best antioxidant profile, given its highest inhibition of DPPH radical (IC_50_ = 6.887 ± 0.151 mg/mL) and ABTS radical scavenging activity (IC_50_ = 19.08 ± 0.02 mg/mL). To the best of our knowledge, more than 88% citral of *C. bodinieri* was investigated and the antioxidant properties described for the first time. Considering high essential oil yield, rich citral content and high antioxidant activity, the N7 variety will be a good candidate for pharmaceutical and cosmetic development of an improved variety.

## 1. Introduction

*Cinnamomum camphora* and *Cinnamomum bodinieri,* from the Lauraceae family, are evergreen broad-leaf trees indigenous to southern China. The chemical polymorphism had been discovered in *C. camphora* and *C. bodinieri*, including linalool-, borneol-, camphor-, cineole-, nerolidol- and citral-types. The citral chemotype *C. camphora* and *C. bodinieri* were so named for the large amount of citral in its root barks, stem barks and leaves. Citral, 3,7-dimethyl-2,6-octadienal, is a precise monoterpenoid widely used in the pharmaceutical and cosmetic industries [1]. It is generally recognized as safe status (GRAS) and listed by the United States Food and Drug Administration (FDA) and hence, when added to food, is considered safe by experts [2]. It is an important chemical raw material for other components’ synthesis, such as ionone, vitamin A, vitamin E, citronitrile, methyl ionone, hydroxyl-citronellal and isohu menthol [3]. As synthetic citral produced highly concentrated waste water, the essential oils extracted from plants meet the demand of people for green natural products [4] and have become commercially popular due to their impression as a “well-being” life style product [5]. Citral, which is a key component of natural plant essential oils and natural antioxidant substances, can inhibit the oxidation of linoleic acid and protect IEC-6 cells against aspirin-induced oxidative stress [6]. It has been increasingly cultivated during the last few years and the world’s interest in citral as an aromatic plant is still increasing.

In the past decade, a considerable body of literature has grown up around the key technologies of the whole industry chain for high-efficiency planting and the intensive processing of *C. camphora* and *C. bodinieri*, including phylogenetic analysis based on the genome of camphor tree [7,8,9], transcriptome analysis and the identification of genes [10,11,12], metabolic pathways and regulatory mechanisms of essential oil biosynthesis [13,14], the effect of exogenous substances during tissue culture [15], comparative extraction method analysis [16], antibacterial, nematicidal and antioxidant activity of essential oil [17,18]; thus, the superior individuals of citral chemotype *Sect. Camphora* species were screened in Nanchang Institute of Technology over the last five years [19] and the optimal rooting medium for the *C. bodinieri* citral type was identified [20]. Nevertheless, the citral chemotype has been a largely under explored domain. The antioxidation of natural plant essential oils is very important for their application in the fields of medicine, food and spices, which overcome the deleterious effect of chemically synthesized antioxidants. The 2,2-diphenyl-1 picrylhydrazyl (DPPH) free radical scavenging test, scavenging 2,2′-azinobis(3-ethylbenzo thiazoline-6-sulfonic acid) diammonium salt radical (ABTS) and the ability of Ferric reducing antioxidant power (FRAP) are the most common methods used to evaluate the antioxidant activity of compounds, and various phytoconstituents and their potential antioxidant activities have been reported previously [21,22,23,24,25,26,27,28]. In terms of the *C. camphora’s* antioxidant activities, linalool, eucalyptol, camphor and borneol chemotypes with strong scavenging activity against 2,2-diphenyl-1 picrylhydrazyl (DPPH) were proved [17]. It is worth noting that the antioxidation of the citral chemotype of *C. camphora* and *C. bodinieri* are still unexplored and need to be clarified.

The objectives of this study are: (1) to select *C. camphora* and *C. bodinieri* with abundant citral accumulation and high essential oil yield under the same growing conditions, which were screened from different geographical regions by our research group in the early stages; (2) to determine the antioxidant activities of the EOCC by DPPH, ABTS and FRAP methods; and (3) to explore the relationship between the terpenoids and antioxidant properties of the EOCC. The results will provide theoretical basis for subsequent plant breeding and intensive utilization of the EOCC.

## 2. Results

### 2.1. Essential Oil Yield

The density of the EOCC was 0.882 ± 0.008 (25 °C) g/cm^3^ and the colors were yellowish (Table 1). The EOCC extracted from N1 and N2 varieties contained cloudy components, the others were transparent. The oil yield of fresh weight and dry weight ranged from 0.6 to 1.11% (*w*/*w*) and from 1.45 to 2.64% (*w*/*w*), respectively. The essential oil yield of different varieties had significant differences according to Duncan’s test with 1% significance (*p* ≤ 0.01) and the *C. camphora* leaves implied a higher essential oil yield than the *C. bodinieri* leaves. The essential oil yields from different geographical origins had no significant difference.

### 2.2. Chemical Constituents of Essential Oil

After integration of the chromatograms and identification of components of seven EOCC with its concentration more than 0.1%, the components were classified by terpene groups (Table 2). The GC-MS experiment identified the N5 variety 94.9% (11 constituents) and N3 variety 94.9% (28 constituents), followed by N7 variety 94.6% (8 constituents), N2 variety 93% (21 constituents), N4 variety 89.2% (22 constituents), N1 variety 88.0% (27 constituents) and N6 variety 87.5% (13 constituents). Monoterpenes (hydrocarbon and oxygenated) dominated in the chemical composition of the N1~N7 EOCC with proportions of 71.7%, 83.2%, 76.3%, 76.7%, 86.1%, 81.8% and 93.9%, respectively. 

**Figure 1:**
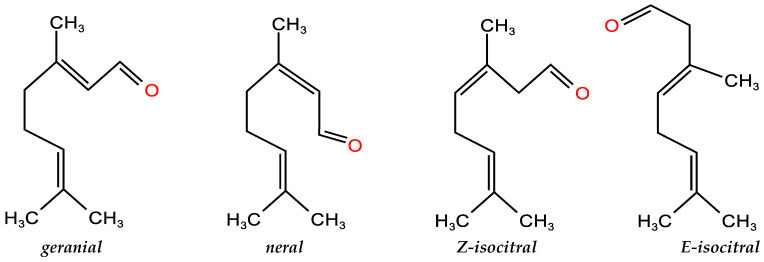
Chemical structures of the main compounds of the essential oils from citral chemotype *C. camphora* and *C. bodinieri*.

Among these compounds, geranial ((2E)-3,7-dimethylocta-2,6-dienal) and neral ((2Z)-3,7-dimethylocta-2,6-dienal) known as citral a and citral b, two geometric isomers of citral (Figure 1), were dominated in the seven EOCCs, which ranged from 60.5% to 88.7%. In particular, the citral of N7 variety was 88.7% (34.6% neral and 54.1% geranial), followed by N5 variety 78.8% (34.1% neral and 44.7% geranial). In addition, the EOCC contained high contents of eucalyptol, sabinene, Z-isocitral, E-isocitral, geraniol, geranic acid, geranyl acetate, caryophyllene, humulene, cyclogermacrene, bicyclogermacrene, 10′-apocarotenonal, humulene epoxide II, etc. 

### 2.3. Antioxidant of the Essential Oil

#### 2.3.1. DPPH Radical Scavenging Activity

**Figure 2:**
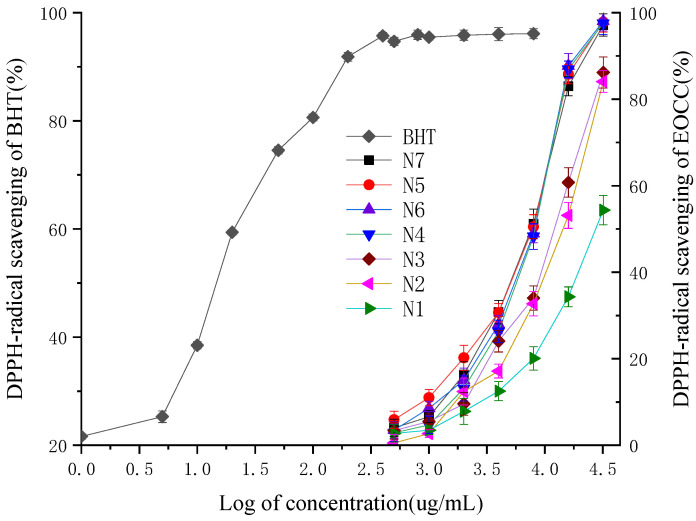
DPPH-radical scavenging activities of the BHT and EOCC.

On the basis of the analysis of the different radical scavenging activity of the EOCC against DPPH between different varieties, the four-parameter logistic curve equation could be established for these relationships to predict the IC_50_ based on log of the EOCC concentration (Table 3), with the 3,5-ditertiobutyl-4-hydroxytoluène (BHT) as a positive control (Figure 2). The coefficients of determination of the logistic curve models of N1, N2, N3, N4, N5, N6, N7 and BHT were elevated to 0.997, 0.996, 0.990, 0.989, 0.987, 0.985, 0.996 and 0.994, respectively, and all F-test values were less than 0.001. The anti-radical activity fell into the following descending order: BHT > N7 > N5 > N6 > N4 > N3 > N2 > N1, with significant differences according to Duncan’s test with 1% significance. The IC_50_ values of seven EOCC were ranged from 6.887 ± 0.151 mg/mL to 28.133 ± 0.44 mg/mL. The most active DPPH radical scavenging activity was N7 (IC_50_ = 6.887 ± 0.151 mg/mL), followed by N5 (IC_50_ = 7.065 ± 0.086 mg/mL) and the lowest DPPH radical scavenging activity was N1 (IC_50_ = 28.133 ± 0.44 mg/mL). The scavenging activity of BHT (IC_50_ = 0.015 ± 0.007 mg/mL) for the DPPH radical was superior to that of EOCC.

The Spearman test revealed a significant negative correlation (*p* ≤ 0.01) between the IC_50_ DPPH and oxygenated monoterpenes (OM) in the EOCC (Table 4). The oxygenated monoterpenes (OM) chemical families that are opposed to the IC_50_ DPPH promote radical scavenging activity against DPPH. The HS (*p* ≤ 0.01) and OS (*p* ≤ 0.05) chemical groups showed high positive correlation coefficients. The HM and NT chemical groups had no effect on the radical scavenging activity against DPPH. 

#### 2.3.2. ABTS Radical Scavenging Activity

**Figure 3:**
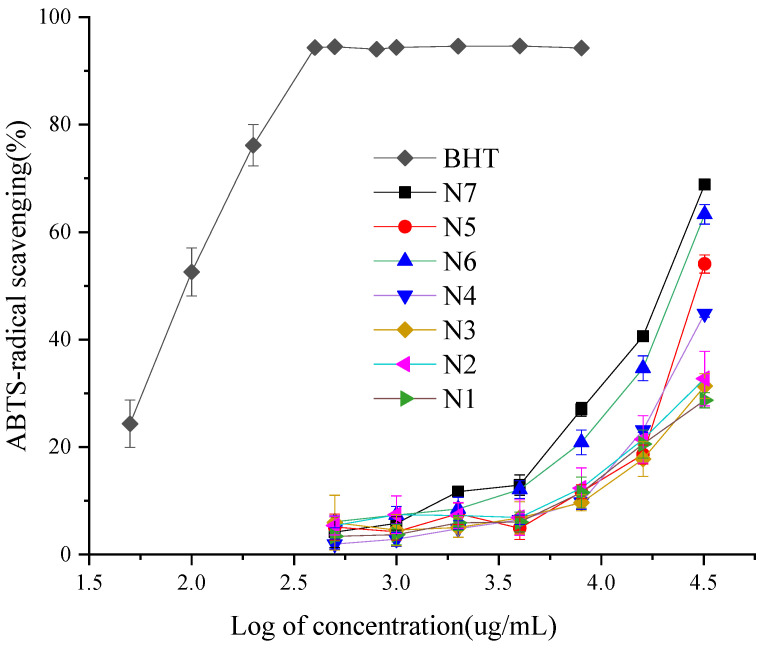
ABTS-radical scavenging activities of the BHT and EOCC.

The analysis of the different ABTS radical scavenging activity of the EOCC, with the 3,5-ditertiobutyl-4-hydroxytoluène (BHT) as a positive control (Figure 3), showed some ABTS radical scavenging activity was dependent on EOCC concentration. The EOCC ABTS radical scavenging capacity fell into the following descending order: BHT > N7 > N6 > N5 > N4 > N2 > N3 > N1, with significant differences according to Duncan’s test with 1% significance (Table 3). The highest ABTS radical scavenging activity was N7 (IC_50_ = 19.08 ± 0.02 mg/mL), followed by N6 (IC_50_ =22.53 ± 0.04 mg/mL) and the lowest ABTS radical scavenging activity was N1 (IC_50_ =117.22 ± 5.4 mg/mL). The scavenging activity of BHT (IC_50_ = 0.10 ± 0.004 mg/mL) for the ABTS radical was superior to that of EOCC. As with the DPPH test, the Spearman test revealed the same rules between ABTS IC_50_ and chemical groups; the ABTS-radical scavenging activities were significantly correlated to oxygenated monoterpenes (Table 4).

#### 2.3.3. Ferric Reducing Antioxidant Power (FRAP)

**Figure 4:**
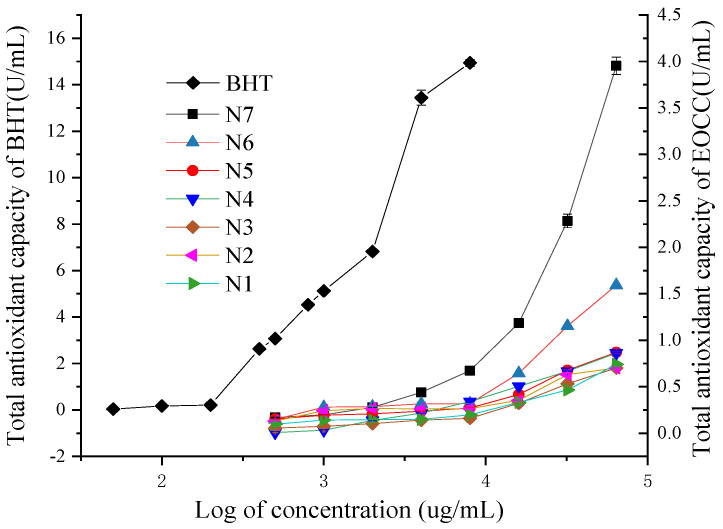
Total antioxidant capacities of the BHT and EOCC.

All EOCCs had some Fe^3+^ reducing capacity and the reduction capacity for Fe^3+^ increased gradually when the concentration of the essential oils increased (Figure 4). Among seven EOCCs, the N7 variety had the highest total antioxidant capacity (T-AOC).

At the same concentration, the T-AOC of the BHT and EOCC fell into the following descending order: BHT > N7 > N6 > N5 > N4 > N1 > N2 > N3. When the EOCC concentration was 64.0 mg/mL, the T-AOCs of N1~N7 were 0.74, 0.71, 0.70, 0.86, 0.87, 1.59, 4.0 U/mL, respectively.

## 3. Discussion

The EOCC oil yields of fresh weight ranging from 0.6 to 1.11% were lower when compared to those identified in the literature for *C. camphora* linalool chemotype (1.3%) [13]; approximately the same result was found in the *Sect. Camphor (Trew.) Meissn*. citral chemotype (0.8%) [19], significantly higher than *C. camphora* ordinary varieties (0.212–0.480%) [16]. The yield of essential oils depends on the genotype [32], geographical origin [33,34], the time of harvest [35], the temperature [36], the humidity level [37], the nature of the soil [38], the organ of the used plant [22,23], the organ’s age [39], plant density [40], nutrient application [32] and the extraction method [16,27,41]. The samples tested in the experiment were collected from the same cutting orchard and the environmental conditions were similar when their leaves were picked. The essential oil yield of different varieties had significant differences (*p* ≤ 0.01) and the *C. camphora* leaves conferred a higher essential oil yield than the *C. bodinieri.* Variations might result from changes in the expression of related genes [32]. *C. bodinieri* leaves are thicker than those of *C. camphora*; thus, in the same process of steam distillation extraction of essential oil, the residual amount of essential oil in the residue is larger, leading to a low oil yield. Therefore, it is necessary to apply other effective technologies for *C. bodinieri*.

The citral (60.5–88.7%) was the main component in the EOCC, equal to or better than the citral-rich plants, including *Backhousia citriodra* (85–95%) [4,26]; *Litsea cubeba* (70–90%) [42]; *Cymbopogan flexuosus* (65–85%) [43]; *Ocimum gratissimum* (65–75%) [44]; *Lippia citriodor* (30–60%) [1,45]; *Citrus aurantium bigarade* (25–30%) [46]. The citral chemotype *C. camphora* and *C. bodinieri*, as the evergreen tree, had more advantages for extracting citral in terms of biomass, oil yield of essential oil and citral content; moreover, their tending and harvesting can be mechanized. Unsaturated aldehydes of the citral structure were quite labile, and iso-citrals as reaction products frequently existed. Z-isocitral and E-isocitral were identified in our research, meanwhile, exo-isocitral was not detected. These marked differences in the citral isomers of *Backhousia citriodra* determined by Southwell et al. [4] from that of the present study could be attributed to species or isomer content difference. After repeated GC-MS experiments to identify the composition of citral chemotype *C. camphora* and *C. bodinieri* essential oil, exo-isocitral was not detected; this result ties in well with previous studies [29,31,47]. We speculated that exo-isocitral content might be less than the GC-MS minimum threshold for detection, resulting in not being identified. 

Four chemotypes of *C. camphora* extracts showed high scavenging activity against DPPH free radicals in July, due to the seasonal variations in the terpenoid content [17], So we chose to conduct this study in July. The four-parameter logistic curve equation could be established for these relationships to predict the IC_50_ and the coefficients of determination of DPPH and ABTS radical scavenging activity were greater than 0.95. The model was well fitted to understand intuitively and predict accurately the IC_50_ value of seven EOCCs. The IC_50_ of BHT in the DPPH test was about 0.015 mg/mL, which is consistent with other reported results (0.012 mg/mL) [48]. The EOCC IC_50_ values were consistent with the IC_50_ of *Cinnamomum parthenoxylon* (4.528 mg/mL) [24], but less than *Cinnamomum iners Reinw. ex Blume* (0.015 mg/mL) [49] and *Lindera pulcherrima* (0.087 mg/mL) [50]. This might be due to the absence of strong biologically active components such as phenols and polyphenols, which have remarkable activity against free radicals. The antioxidant activity of EOCC is significantly inferior to that of BHT, but the plant essential oil is a natural substance with the advantage of being green, clean, environmentally friendly and of good potential application.

The essential oil exhibited strong concentration dependency in a sigmoidal dose-response curve over the concentration range. Other studies on the antioxidants of essential oils have proved that the DPPH and ABTS radical scavenging ability of essential oils exhibits a significant positive correlation with the concentration of essential oils and has a close connection with its chemical components, especially its main components [28]. In the DPPH, ABTS and FRAP assays, although their ranking differed slightly, all assays identified the top three varieties according to their antioxidant capacities as N7, N5 and N6 varieties. This could be due to the synergetic effects of the identified essential oil components. DPPH radicals can be scavenged because essential oils donate a hydrogen atom to DPPH and give rise to the reduced DPPH-H with the loss of this violet color [51]. The main components of the EOCC are oxygenated terpenoids such as neral and geranial, which have a great impact on the antioxidant activity of essential oil. According to the classification and analysis of the main components of the EOCC, the antioxidant activity of the essential oils is positively correlated with the content of oxygenated terpenoids (oxygenated monoterpenes and sesquiterpenes), due to terpenoid antioxidant activity depending on the numbers and positions of C=C double bonds [52], which can easily react with free radicals and ROS to serve their antioxidant function. Terpenoids have also been found to possess chain-breaking antioxidant activity [17,53], which are similar to phenols. A previous study found that the strongest scavenging activity was mainly detected in the *C. camphora* extracts, which had the highest terpenoid content among the four chemotypes [17]. The molecular mechanism of the EOCC radical scavenging activity had been a largely under-explored domain. We wish to extend this study to the relationship between the counterpart compositions of *C. camphora* and *C. bodinieri* and their radical scavenging activity. 

## 4. Materials and Methods

### 4.1. Plant Material and Reagent

Healthy pest-free mature leaves (200 g) of *C. camphora* and *C. bodinieri* were harvested from 5-year-old clones grown at the cuttings orchard of NanChang Institute of Technology in July 2022 (Latitude: 28°41′47″ N, Longitude: 116°1′49″ W). The clones were propagated from mother trees through cutting propagation. For each biological replicate (*n* = 3), leaves from at least six tree clones, which were cloned from the same mother tree, were collected from the east, south, west and north of the canopy, and mixed. Afterward, the leaves were stored at 4 °C until isolation. The citral-rich asexual mother plants were collected from 40,000 *C. camphora* and *C. bodinieri* in their natural geographic distributions, included Jiangxi, Guangxi, Hubei and Guizhou, ranging from 2011 to 2017, and propagated into the cuttings orchard of NanChang Institute of Technology in 2017. The plants were authenticated by Professor Zhinong Jin. The voucher specimens were deposited in the Gene Bank of the Camphor Tree laboratory, Jiangxi Provincial Engineering Research Center For Seed-Breeding and Utilization of Camphor Trees, and the voucher numbers were for *C. camphora*-GX/ZS/004 (N1 variety); *C. bodinieri*-GX/QZ/003 (N2 variety); *C. camphora*-GX/ZS/003 (N3 variety); *C. camphora*-JX/NC/002 (N4 variety); *C. camphora*-JX/NC/001 (N5 variety); *C. bodinieri*-HB/CY/021 (N6 variety); *C. bodinieri*-GZ/TZ/028 (N7 variety).

The 2,2-Diphenyl-1-picrylhydrazyl (DPPH), Methanol and 6-Di-tert-butyl-4-methyl phenol (BHT) were purchased from Macklin Reagent Co., (Shanghai, China, www.macklin.cn, accessed on 14 January 2022). CO_2_ (99.5 wt.%) was purchased from Hongqing Gas Co., (Nanchang, China). Citral, 2,2′-azinobis (3-ethylbenzothiazoline-6-sulfonic acid ammonium salt) (ABTS), potassium persulfate and C7-C40 saturated alkanes standard were obtained from Shanghai Aladdin (Shanghai, China, www.aladdin-e.com, accessed on 8 July 2022). Ethanol was procured by Sinopharm Chemical Reagent Co., Ltd. (Shanghai, China, www.reagent.com.cn, accessed on 24 February 2022). Total antioxidant capacity (T-AOC) diagnostic kits were obtained from Sangon Biotech (Shanghai, China). All reagents were AR grade.

### 4.2. Isolation of Essential Oil

Leaves (200 g) were placed into a 1000 mL extraction stainless steel cell for oil extraction immediately after harvesting. Leaf samples were hydro-distilled in a modified Clevenger apparatus (the patent application number: 201710158988.8) for 90 min. The essential oil was dried over anhydrous sodium sulphate separately and kept in a refrigerator (4 °C) for GC-MS [19].

Extracted essential oil was weighed, and the rate of water content was measured by MA150 rapid moisture analyzer (Sartorius, Germany), which was repeated three times. Finally, the oil yield was calculated using the formula:

Fresh leaf essential oil yield (%) = *W*_1_/*W*_2_ × 100

Dry leaf essential oil yield (%) = *W*_1_/(*W*_2_ × (100% − *M*)) × 100

where *W*_1_ is the weight of extracted essential oil; *W*_2_ is the weight of fresh leaves; and *M* is rate of water content.

### 4.3. Gas Chromatography-Mass Spectrometry (GC-MS)

Analyses of essential oils were performed on a gas chromatography system (Agilent 7890B-5975C GC-MS; USA) equipped with a Agilent J&W HP-5MS column (30 m × 250 μm × 0.25 μm). Referring to the experimental conditions of our previous study, the mass spectra electronic impact was taken at 70 eV, the scanned mass range was set at 50 to 650 m/z, the scanned rate was set at 0.5 scans/s, the conductor temperature was 250 °C, the ion source temperature was 230 °C, the quadrupole temperature was 150 °C and the multiplier voltage was 1200 V. Helium was the carrier gas (flow rate of 2.6953 mL/min) and an injection volume of 0.1 µL was employed (split ratio 20:1). Oven temperature program conditions were as follows: initial temperature of 80 °C for 5 min with a solvent delay of 3 min, then gradually increased to 120 °C at a 2.5 °C/min rate, where it remained for 1 min, then ramped at 20 °C/min to 240 °C for 5 min, total run time 60 min. Essential oils were diluted with methanol (1%), filtered and injected manually. 

The chemical compounds’ data of the essential oils were exported using the supplied enhanced data analysis software, selecting the Wiley7n.l /NIST17.L library of spectra; the citral standards were used as controls to find the corresponding compounds according to the comparison of their relative retention time (RT). Retention indices (RI) were measured with respect to C7-C40 saturated alkanes standard.

### 4.4. Antioxidant Activity DPPH Test

The effects of 1,1-diphenyl-2-picrylhydrazyl (DPPH) free radical scavenging potentials of the essential oils were determined on the basis of the method described by Brand-Williams et al. [54], prepared with some modifications. A total of 3.94 mg (0.01 mmol) of DPPH were dissolved in 100 mL of ethanol. The 0.1 mmol/L DPPH solution (2.0 mL) was mixed with 2 mL of essential oils of 32.0, 16.0, 8.0, 4.0, 2.0, 1.0 and 0.5 mg/mL. The absorbance reading for each concentration was taken at 517 nm after 30 min of incubation in the dark at room temperature. The 6-Di-tert-butyl-4-methylphenol (BHT) was used as a positive control and ethanol was measured as a negative control. All spectrophotometric data were acquired using a Molecular Devices SpctraMax 190 (USA). The analyses were performed in 3 replications.

The antioxidant activity linked to inhibition percentage of DPPH was calculated by the equation: Inhibition (%) = (*A*_0_ − *A*_1_)/*A*_0_ × 100%, where *A*_0_ is ethanol DPPH blank absorbance, *A*_1_ is sample DPPH absorbance.

The radical scavenging activity of the studied samples was expressed as IC_50_, defined as the concentration of the essential oil necessary to reduce or inhibit 50% of DPPH radical solution. The best activity against the DPPH radical was obtained with the lowest value of IC_50_. IC_50_ were estimated from the inhibition percentage versus concentration plots using a non-linear regression algorithm.

### 4.5. ABTS Radical Scavenging Activity

The ABTS^+^ was produced by reacting 1:1 substance ratio 7 mmol/L stock solution of ABTS with 2.45 mmol/L potassium persulfate and allowing the mixture to stand in the dark for 12–16 h at room temperature. After incubation, the solution ABTS^+^ was diluted with methanol to obtain an absorbance of 0.70 ± 0.02 at 734 nm. A volume of 0.2 mL of essential oil at the tested concentration (64.0, 32.0, 16.0, 8.0, 4.0, 2.0, 1.0 and 0.5 mg/mL) was added to 3.8 mL of the ABTS^+^ solution. Absorbance was measured at 734 nm. The percentage inhibition of the radical cation ABTS^+^ was determined using the following formula: Inhibition of ABTS (%) = (*A*_0_ − *A*_1_)/*A*_0_ × 100%, where *A*_0_ is ethanol ABTS^+^ blank absorbance, *A*_1_ is the essential oil absorbance.

### 4.6. Ferric Reducing Antioxidant Power (FRAP)

FRAP was measured by total antioxidant capacity (T-AOC) diagnostic kits (Shanghai, China). The FRAP reagent 1, 2, 3 were mixed daily at the volume ratio of 7:1:1. A total of 180 uL FRAP reagent, 18 uL double distilled water were mixed in 1 mL centrifugal tube with 6 uL of essential oil solution (64.0, 32.0, 16.0, 8.0, 4.0, 2.0, 1.0 and 0.5 mg/mL). The mixture was vigorously shaken, and absorbance was measured at 593 nm after 10 min.

Ferrous sulfate standard solution (40 umol/mL) was produced by reacting 10 mg ferrous sulfate heptahydrate, 0.9 mL distilled water and 20 uL concentrated sulfuric acid. The standard solution was diluted to 0.15, 0.1, 0.05, 0.025, 0.0125, 0.00625, 0.003125, 0.00156 umol/mL, then mixed with 100 uL standard solution and 100 uL TPTZ solution. Absorbance was measured at 593 nm after 10 min. All measurements were repeated 3 times. Total antioxidant capacity in the measuring systems, expressed as ferrous sulfate equivalents, was calculated. Correlation coefficient (R^2^) for the calibration curve was 0.9982.

The total antioxidant capacity (U/mL) = *X* × *V_t_*/*V_s_*, where *X* is the sample antioxidant capacity expressed as the concentration of the FeSO_4_ solution when the absorbance of the sample is equal to the absorbance of the FeSO_4_ standard solution (umol/mL), *V_t_* is 0.204 mL, *V_s_* is 0.006 mL.

### 4.7. Statistical Analysis

All data represent the mean of 3 tests ± standard deviations (SD). Analysis of variance (ANOVA) test was conducted using SPSS 22.0. Origin 2018 software (Origin Lab, Northampton, MA, USA) was used for graphical analysis. GraphPad Prism (GraphPad Software 8.0.1) was used for IC_50_. KingDraw chemical structure editor software was used to depict the chemical structure.

## 5. Conclusions

In this paper, we studied the oil yield, essential oil composition and antioxidant activities of seven citral chemotype *C. camphora* and *C. bodinieri* of different origins and conducted a comparative analysis to explore the relationship between their antioxidant activities and their main components. The main component of the essential oil was citral (neral and geranial), with GC-MS concentrations ranging from 60.5–88.7%. The N7 variety had the highest citral content in seven EOCCs and the antioxidant activity was significantly stronger than other varieties in the DPPH, ABTS and FRAP assays, therefore, it could be preferentially selected as the raw material for the extraction of citral. The seven essential oils had a moderate antioxidant capacity, showing a positive correlation with the content of oxygenated terpenoids in the EOCC. This study made a major contribution by identifying that the citral chemotype *C. bodinieri* is an unrivalled source of citral by demonstrating large biomass, high oil yield and rich citral content.

## Figures and Tables

**Table 1:** The essential oil yield and characteristics of citral chemotype *C. camphora* and *C. bodinieri*.

Varieties	Geographical Origins	Species	Density (g/cm^3^)	Percentage Yield %		Essential Oil Characteristics
				Fresh Weight	Dry Weight	
N1	Guangxi	*C. camphora*	0.887 ± 0.003	1.02 ± 0.05 ^a^	2.34 ± 0.17 ^ab^	Cloudy and yellowish
N2	Guangxi	*C. bodinieri*	0.868 ± 0.005	0.77 ± 0.06 ^b^	1.78 ± 0.15 ^cd^	Cloudy and yellowish
N3	Guangxi	*C. camphora*	0.880 ± 0.016	1.11 ± 0.07 ^a^	2.64 ± 0.15 ^a^	Transparent and yellowish
N4	Jiangxi	*C. camphora*	0.894 ± 0.001	0.82 ± 0.06 ^b^	2.04 ± 0.14 ^bc^	Transparent and yellowish
N5	Jiangxi	*C. camphora*	0.879 ± 0.021	1.08 ± 0.04 ^a^	2.60 ± 0.17 ^a^	Transparent and yellowish
N6	Hubei	*C. bodinieri*	0.886 ± 0.010	0.60 ± 0.09 ^c^	1.45 ± 0.23 ^d^	Transparent and yellowish
N7	Guizhou	*C. bodinieri*	0.884 ± 0.002	0.83 ± 0.05 ^b^	2.04 ± 0.12 ^bc^	Transparent and yellowish

Note: lowercase letters compare data within each sample. Data followed by the different letters indicate significant differences according to Duncan’s test with 1% significance (*p* ≤ 0.01).

**Table 2:** Essential oil composition of citral chemotype *C. camphora* and *C. bodinieri*.

No	RI ^a^ (lit)	RI ^b^ (exp)	Compounds ^c^	Molecular Formula	Percent Composition						
					N1	N2	N3	N4	N5	N6	N7
1	972	975	sabinene	C_10_H_16_	-	6.0	-	-	-	-	-
2	981	978	β-Pinene	C_10_H_16_	-	-	0.4	0.3	-	-	-
3	1026	1025	limonene	C_10_H_16_	-	-	-	-	-	-	0.2
4	1030	1029	eucalyptol	C_10_H_18_O	1.1	4.5	0.1	-	-	-	-
5	1101	1098	linalool	C_10_H_18_O	-	2.0	-	-	-	-	0.3
6	1158	1156	citronellal	C_10_H_18_O	-	-	-	-	-	-	1.5
7	1165	1165	Z-isocitral	C_10_H_16_O	3.2	3.2	3.1	-	-	1.8	-
8	1184	1179	E-isocitral	C_10_H_16_O	3.8	3.5	3.2	3.9	4.7	-	-
9	1227	1217	nerol	C_10_H_18_O	1.2	-	-	-	-	1.1	-
10	1245	1247	neral	C_10_H_16_O	28.7	28.6	32.6	30.7	34.1	39.2	34.6
11	1254	1244	geraniol	C_10_H_18_O	2.0	-	-	1.5	2.6	1.9	1.3
12	1276	1277	geranial	C_10_H_16_O	31.8	35.3	36.9	40.2	44.7	35.3	54.1
13	1355	1346	geranic acid	C_10_H_16_O_2_	-	-	0.1	0.1	-	2.6	2.0
14	1354	1353	β-Citronellyl acetate	C_12_H_22_O_2_	0.3	-	0.6	0.4	-	-	-
15	1362	1361	neryl acetate	C_12_H_20_O_2_	-	-	0.3	-	-	-	-
16	1371	1370	α-copaene	C_15_H_24_	-	-	0.2	-	-	-	-
17	1388	1380	β-elemene	C_15_H_24_	0.3	0.2	-	-	-	-	-
18	1384	1383	geranyl acetate	C_12_H_20_O_2_	0.6	0.1	4.1	0.8	-	-	-
19	1410	1403	caryophyllene	C_15_H_24_	1.9	1.6	2.2	1.0	2.4	-	0.6
20	1454	1454	humulene	C_15_H_24_	1.0	0.5	1.3	1.0	-	-	-
21	1480	1477	germacrene D	C_15_H_24_	0.3	0.3	0.2	0.3	-	0.3	-
22	1486	1484	β-selinene	C_15_H_24_	0.7	0.2	0.4	0.6	-	-	-
23	1495	1491	bicyclogermacrene	C_15_H_24_	2.2	2.8	0.8	2.1	2.6	0.7	-
24	1524	1518	delta-cadinene	C_15_H_24_	-	-	0.2	-	-	-	-
25	1537	1540	elemol	C_15_H_26_O	0.2	0.1	0.4	0.4	0.5	-	-
26	1550	1548	(1E,5E)-germacrene B	C_15_H_24_	0.1	0.1	0.1	0.1	-	-	-
27	1562	1557	(E)-nerolidol	C_15_H_26_O	0.2	0.1	0.3	1.6	1.5	-	-
28	1577	1571	10′-apocarotenal	C_15_H_24_O	2.9	-	3.9	0.9	0.6	2.8	-
29	1576	1572	spathulenol	C_15_H_24_O	0.4	3.2	0.5	0.3	-	0.3	-
30	1578	1574	caryophyllene oxide	C_15_H_24_O	2.7	-	0.1	1.1	0.8	-	-
31	1591	1587	guaiol	C_15_H_26_O	0.3	0.2	-	0.3	0.4	-	-
32	1593	1589	humulene oxide I	C_15_H_24_O	-	-	0.3	-	-	-	-
33	1606	1600	humulene epoxide II	C_15_H_24_O	1.2	0.2	1.5	1.4	-	0.2	-
34	1631	1624	caryophylla-4(12),8(13)-dien-5.alpha.-ol	C_15_H_24_O	0.1	-	-	-	-	-	-
35	1635	1635	τ-cadinol	C_15_H_26_O	-	-	0.2	-	-	-	-
36	1643	1645	τ-muurolol	C_15_H_26_O	-	0.3	-	-	-	-	-
37	1651	1647	selin-11-en-4-α-ol	C_15_H_26_O	0.5	-	0.7	-	-	0.3	-
38	1705	1705	(Z,Z)-2,6-farnesol	C_15_H_26_O	0.2	-	0.2	0.2	-	-	-
39	1709	1709	(Z)-epi-β-santalol	C_15_H_24_O	-	-	-	-	-	1.0	-
40	1720	1721	(E,Z)-2,6-farnesal	C_15_H_24_O	0.1	-	-	-	-	-	-
Amount of chemical compounds					27	21	28	22	11	13	8
Total identified constituents					88.0	93.0	94.9	89.2	94.9	87.5	94.6
Hydrocarbon monoterpenes (HM) 1,2,3.					0.0	6.0	0.4	0.3	0.0	0.0	0.2
Oxygenated monoterpenes (OM) 4,5,6,7,8,9,10,11,12,13.					71.7	77.2	75.9	76.4	86.1	81.8	93.7
Hydrocarbon sesquiterpenes (HS) 16,17,19,20,21,22,23,24,26.					6.4	5.6	5.1	5.0	5.0	1.0	0.6
Oxygenated sesquiterpenes (OS) 25,27,28,29,30,31,32,33,34,35,36,37,38,39,40.					8.8	4.2	8.0	6.1	3.7	4.5	0.0
Non-terpenic compounds (NT) 14,15,18.					0.9	0.1	0.6	1.2	0.0	0.0	0.0

Note: Table presents only the compounds for which the peak area exceeds 0.1% in chromatograms of the analyzed EOCC. a. RI (lit) refers to “Retention Index” found in the Wiley7n.l /NIST17.L library and the literature [29,30,31]; b. RI (exp) refers to “Retention Index” measured with respect to saturated alkanes standard; c. Compounds listed in the order of elution from a,—not detected.

**Table 3:** Scavenging activity of the EOCC against DPPH and ABTS free radicals.

No	DPPH			ABTS		
	IC_50_ (mg/mL)	Fitting Equation	R^2^	IC_50_ (mg/mL)	Fitting Equation	R^2^
N1	28.133 ± 0.44 a	$y=864.15-\frac{863.48}{1+{\left(\frac{x}{6.58}\right)}^{7.16}}$	0.997	117.22 ± 5.4 a	$y=34.63-\frac{30.82}{1+{\left(\frac{x}{14.57}\right)}^{1.81}}$	0.995
N2	14.504 ± 0.40 b	$y=1175.97-\frac{1179.24}{1+{\left(\frac{x}{6.59}\right)}^{6.64}}$	0.996	57.33 ± 0.08 c	$y=109.48-\frac{104.09}{1+{\left(\frac{x}{72.97}\right)}^{1.19}}$	0.988
N3	12.229 ± 0.169 c	$y=220.79-\frac{219.83}{1+{\left(\frac{x}{4.76}\right)}^{8.24}}$	0.990	66.9 ± 0.13 b	$y=74.22-\frac{69.75}{1+{\left(\frac{x}{43.77}\right)}^{1.47}}$	0.999
N4	7.527 ± 0.106 d	$y=109.97-\frac{106.14}{1+{\left(\frac{x}{3.92}\right)}^{16.46}}$	0.989	37.87 ± 0.06 d	$y=75.59-\frac{71.38}{1+{\left(\frac{x}{27.65}\right)}^{1.85}}$	0.999
N5	7.065 ± 0.086 e	$y=116.55-\frac{109.01}{1+{\left(\frac{x}{3.97}\right)}^{13.42}}$	0.987	29.91 ± 0.06 de	$y=77.63-\frac{71.22}{1+{\left(\frac{x}{25.60}\right)}^{2.93}}$	0.986
N6	7.371 ± 0.067 de	$y=116.88-\frac{111.3}{1+{\left(\frac{x}{3.99}\right)}^{14.11}}$	0.985	22.53 ± 0.04 e	$y=84.44-\frac{77.73}{1+{\left(\frac{x}{19.81}\right)}^{1.78}}$	0.997
N7	6.887 ± 0.151 e	$y=116.85-\frac{113.55}{1+{\left(\frac{x}{3.95}\right)}^{12.49}}$	0.996	19.08 ± 0.02 e	$y=88.97-\frac{83.44}{1+{\left(\frac{x}{17.47}\right)}^{1.51}}$	0.991
BHT	0.015 ± 0.007 f	$y=99.44-\frac{78.37}{1+{\left(\frac{x}{1.35}\right)}^{3.72}}$	0.994	0.10 ± 0.004 f	$y=94.31-\frac{70.45}{1+{\left(\frac{x}{0.11}\right)}^{4.92}}$	0.870

Note: The lowercase letters correspond to the significant difference among the IC_50_ DPPH and IC_50_ ABTS of the essential oils. Data followed by the different letters indicate significant differences according to Duncan’s test with 1% significance (*p* ≤ 0.01).

**Table 4:** Matrix of correlations between IC_50_ DPPH and chemical composition of citral chemotype *C. camphora* and *C. bodinieri*.

	HM	OM	HS	OS	NT	DPPH	ABTS
HM	1.000						
OM	−0.185	1.000					
HS	0.243	−0.811 *	1.000				
OS	0	−0.964 **	0.667	1.000			
NT	0.346	−0.852 *	0.617	0.778 *	1.000		
DPPH	0.296	−0.893 **	0.937 **	0.786 *	0.704	1.000	
ABTS	0.259	−0.929 **	0.955 **	0.821 *	0.741	0.929 **	1.000

Note: HM: hydrocarbon monoterpenes; OM: oxygenated monoterpenes; HS: hydrocarbon sesquiterpenes; OS: oxygenated sesquiterpenes; NT: non terpenic compounds; DPPH: IC_50_ value in DPPH test; ABTS: IC_50_ value in ABTS assay, * *p* ≤ 0.05, ** *p* ≤ 0.01.

## Data Availability

Not applicable.
